# Recombinant ADAMTS-13 Improves Survival of Mice Subjected to Endotoxemia

**DOI:** 10.3390/ijms241411782

**Published:** 2023-07-22

**Authors:** Daniel Gao, Zhou Zhou, Ruidong Ma, Huaizhu Wu, Trung Nguyen, Li Liu, Jingfei Dong

**Affiliations:** 1 Bloodworks Research Institute, Seattle, WA 98102, USA; dlga2021@mymail.pomona.edu (D.G.);; 2 Department of Chemistry, Pomona College, Claremont, CA 91711, USA; 3 Cardiovascular Sciences, Department of Medicine, Baylor College of Medicine, Houston, TX 77030, USA; 4 Division of Critical Care Medicine, Department of Pediatrics, Baylor College of Medicine, Houston, TX 77030, USA; 5 Center for Translational Research on Inflammatory Diseases at the Michael E. DeBakey Veteran Administration Medical Center, Houston, TX 77030, USA; 6 Tianjin Neurology Research Institute, Tianjin Medical University General Hospital, Tianjin 300052, China; 7 Division of Hematology, Department of Medicine, University of Washington School of Medicine, Seattle, WA 98195, USA

**Keywords:** ADAMTS-13, platelets, von Willebrand factor, endotoxemia, endotheliopathy

## Abstract

When stimulated by proinflammatory mediators, endothelial cells release ultra-large von Willebrand factor (ULVWF) multimers that are hyperactive in activating and aggregating platelets. These ULVWF multimers can accumulate in the circulation and on the inflamed endothelium because they are insufficiently cleaved by the metalloprotease ADAMTS-13, which becomes moderately deficient under conditions of systemic inflammation. This moderate ADAMTS-13 deficiency may lead to thrombotic complications that contribute to ischemic tissue injury and organ failure that are associated with severe infections. To test this hypothesis, we investigated whether recombinant ADAMTS-13 improves the pathological course of endotoxemia in lipopolysaccharide (LPS)-treated mice. C57BL/J6 mice received a bolus infusion of either 5 µg/mouse of ADAMTS-13 or vehicle control 30 min after LPS challenge and were monitored for seven-day survival. During the monitoring period, platelet counts, VWF antigen, and ADAMTS-13 activity were measured. Thrombosis was also examined by the immunohistochemistry in the liver. We found that ADAMTS-13 reduced mortality from 66% to 34.9%. The improved survival was associated with a greater recovery from thrombocytopenia, higher plasma ADAMTS-13 activity, and less thrombotic vascular occlusion. These results suggest that systemic inflammation could result in deficient ULVWF proteolysis by ADAMTS-13 and that ADAMTS-13 improves the outcomes of endotoxemia-induced inflammation.

## 1. Introduction

A hallmark event of systemic inflammation is the activation of endothelial cells, resulting in the release of molecules, which propagate inflammation and lead to ischemic tissue injury and organ failure. Among these molecules is the adhesion ligand von Willebrand factor (VWF) [1]. VWF mediates platelet adhesion to the subendothelium at sites of vessel injury, a crucial step of hemostasis. It also aggregates and activates platelets in the circulation, resulting in thrombotic vascular occlusion and consumptive thrombocytopenia. Despite being a well-known marker for endothelial injury, the role of VWF in propagating inflammation and associated complications remains poorly understood.

VWF is synthesized in endothelial cells and megakaryocytes as pro-VWF monomers. These monomers first form homodimers through C-terminal disulfide bonds, and the dimers are then multimerized through N-terminal disulfide bonds [2]. The newly synthesized VWF multimers are either constitutively released or targeted for storage in the Weibel-Palade bodies of endothelial cells and the α-granules of megakaryocytes/platelets [3]. The stored VWF multimers are abundant in the ultra-large (UL) forms, which are very adhesive and capable of binding the platelet GP Ib–IX–V complex with higher-strength bonds and spontaneously aggregating platelets [4]. The stored ULVWF multimers are released in response to traumatic and inflammatory insults [5]. Upon release, ULVWF multimers undergo rapid but limited proteolysis by the metalloprotease ADAMTS-13 that converts the prothrombotic ULVWF multimers to smaller but hemostatically active forms [6,7]. The cleavage, which takes place on the surface of endothelial cells [6], occurs at the peptide bond between Tyr1605 and Met1606 in the A2 domain of VWF [8]. Severe ADAMTS-13 deficiency, due to genetic mutations or autoantibodies against the metalloprotease, is associated with thrombotic thrombocytopenic purpura (TTP) [9,10,11]. However, mild-to-moderate ADAMTS-13 deficiency was also reported in conditions such as post-surgery, pregnancy-related pathologies (e.g., pre-eclampsia and HELLP syndrome), trauma, and bacterial and viral infections [12,13,14,15,16,17,18]. Although diverse in their causes, these conditions share the common characteristics of systemic inflammation. Even with healthy individuals, a bolus injection of LPS or desmopressin, which induces the systemic release of ULVWF from endothelial cells [19], results in transient ADAMTS-13 deficiency [20,21]. The ADAMTS-13 deficiency found in systemic inflammation could result from a combination of increased consumption, reduced synthesis, proteolytic clearance, and the presence of inhibitors to the metalloprotease [21,22]. A key question is whether this moderate deficiency is pathophysiologically important. If the answer is yes, one would expect that the supplementation of exogenous ADAMTS-13 is protective against such complications of severe systemic inflammation. To test this hypothesis, we studied how recombinant ADAMTS-13 affects the pathological course of systemic inflammation in a mouse model of endotoxemia.

## 2. Results

### 2.1. rhADAMTS-13 Had a Relatively Slow Rate of Clearance

**Figure 1:**
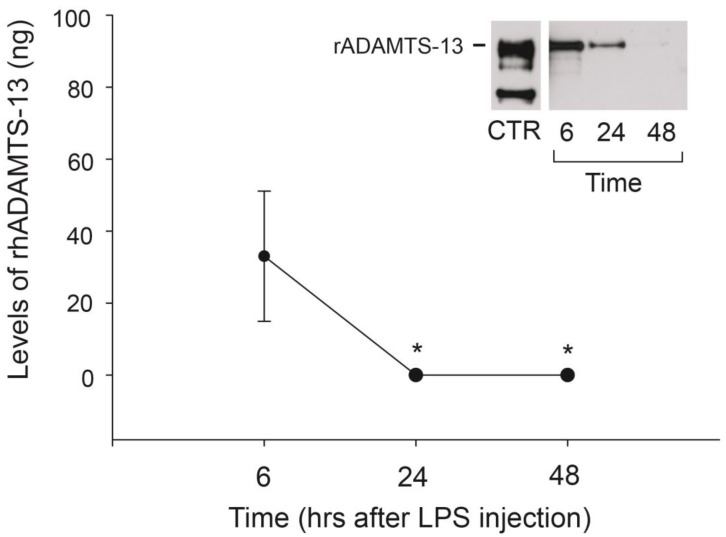
Clearance of rhADAMTS-13 in mouse circulation: blood was collected from the eyes 6, 24, and 48 h after rhADAMTS-13 infusion. Plasma levels of the metalloprotease were measured by capturing ELISA (n = 6, one-way ANOVA, * *p* < 0.05, compared to the value measured 6 h post-ADAMTS-13 infusion) and immunoblots (insert, n = 4).

To determine the clearance rate of recombinant human ADAMTS-13 (rhADAMTS-13), we collected blood samples from non-treated mice 6, 24, and 48 h after they were infused with the metalloprotease. After 6 h, rhADAMTS-13 was detectable by ELISA (20 µL of plasma) and immunoblot (100 µL of plasma), but it was detectable only by immunoblot after 24 h (Figure 1). This is likely because a larger volume of blood was used for immunoblotting (100 vs. 20 µL of blood). The calculated amounts of circulating rhADAMTS-13 after 6 and 24 h were 2.35 µg/mL and 1.6 µg/mL, accounting for 47% and 32%, respectively, of the originally infused 5 µg. rhADAMTS-13 was not detectable 48 h after injection by either assay, indicating that it was largely removed from circulation.

### 2.2. Infusion of rhADAMTS-13 Improved Survival of Mice with Endotoxemia

A total of 117 mice with comparable age and body weight were analyzed (Table 1). Among them, 93 were studied for seven-day survival, and the rest were processed for laboratory tests and immunohistochemistry.

**Figure 2:**
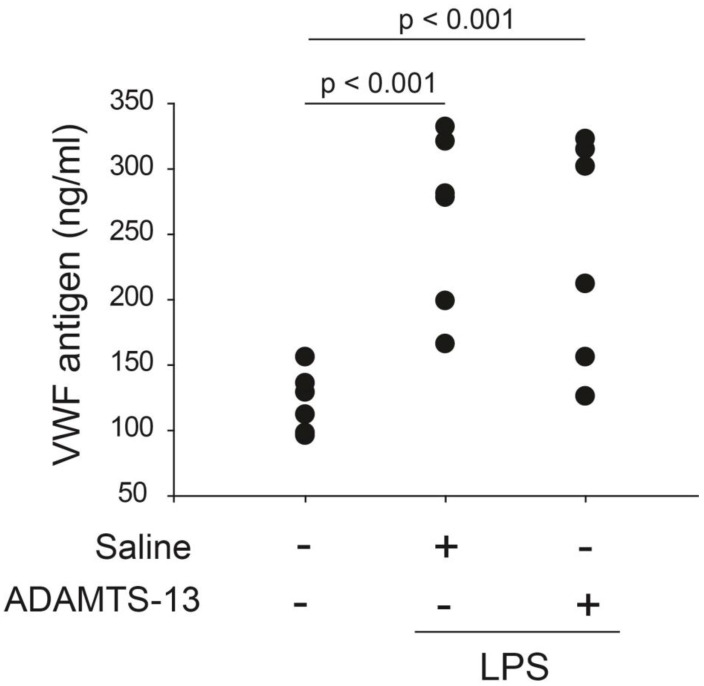
Plasma VWF levels: plasma VWF was measured by a commercial ELISA kit 24 h after LPS challenge. LPS resulted in a significant increase in plasma VWF antigen. The increase was similar for mice receiving rhADAMTS-13 and those receiving vehicle control (n = 6/group, one-way ANOVA, n = 28).

Plasma VWF was first measured after the background reading from the non-specific binding of the control IgG was subtracted in a subgroup of mice to gauge the levels of LPS-induced endothelial cell injury and the rate of VWF secretion. Plasma VWF antigen was significantly increased 6 h after LPS injection, and the increase was similar for mice receiving rhADAMTS-13 and those receiving vehicle control (Figure 2).

**Figure 3:**
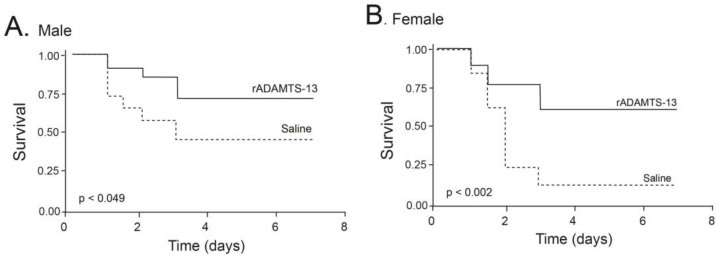
Seven-day survival of endotoxemic mic: C57BL/6J mice were infused with either 5 µg of rhADAMTS-13 or an equal volume of vehicle control 30 min before being injected with 6 mg/kg of LPS. Survival was monitored for seven days and evaluated by Kaplan–Meier survival analysis stratified by males (**A**) and females (**B**) (n = 93 with 52 males and 41 females).

For the survival studies, C57BL/J6 mice were injected with LPS (6 mg/kg) 30 min before they were infused with either rhADAMTS-13 or an equal volume (100 µL) of vehicle control. They were then monitored for seven days under normal feeding conditions. By the end of the seventh day, 66% (33/50) of mice died in the control group, significantly higher than 34.9% (15/43) of mice in the group of mice receiving rhADAMTS-13. The mortality reduction was greater in female mice than that of male mice (Figure 3). However, the mortality rate appeared to be similar for mice receiving rhADAMTS-13 and those receiving vehicle control when a higher dose (20 mg/kg) of LPS was given).

### 2.3. Infusion of rhADAMTS-13 Improved Thrombocytopenia

**Figure 4:**
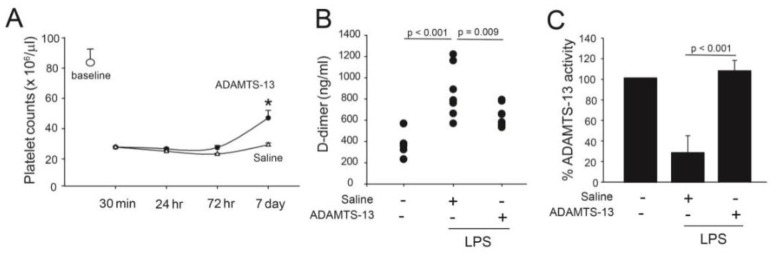
Platelet counts, D-dimer, and ADAMTS-13 activity in endotoxemic mice: (**A**) platelet counts were measured on an ABX Micros 60 Human Blood analyzer 30 min, 24 h, 72 h, and 7 days after LPS injection (n = 10/group, student’s t-test, * *p* < 0.05). (**B**) Plasma levels of D-dimer measured at 72 h post-LPS treatment (n = 10/group, one-way ANOVA). (**C**) ADAMTS-13 activity was measured by a flow-based assay. Plasma from surviving LPS-treated mice at the end of 7 days was mixed with human platelets and perfused over histamine-activated HUVECs for 2 min at 2.5 dynes/cm^2^. The number of ULVWF strings remaining at the end of perfusion was counted (n = 10/group, one-way ANOVA).

The mean platelet count was 8.2 ± 5.4 × 10^5^/µL for untreated mice. LPS induced severe thrombocytopenia that lasted for ~48 h before steadily improving. For surviving mice, the platelet counts on day 7 post-LPS injection increased to ~60% of the baseline level. On day 7, the increase in platelet counts was significantly greater in mice receiving rhADAMTS-13 compared with those receiving vehicle control (Figure 4A). Mice receiving LPS also developed a hyperfibrinolytic state defined by the increased plasma levels of the fibrinolytic product D-dimer, which was also improved in mice receiving LPS and rhADAMTS-13 (Figure 4B).

### 2.4. Endotoxemia Decreased ADAMTS-13 Activity

Due to the difficulties in collecting and culturing mouse vascular endothelial cells, we used human ULVWF strings to determine the ADAMTS-13 activity of mouse plasma in a flow-based activity assay [6]. In this system, mouse plasma cleaved human ULVWF strings at ~70–80% of human plasma activity. This cross-activity between human and mouse ADAMTS-13 was previously reported [23]. Using this assay, we found that ULVWF-cleaving activity was significantly lower in LPS-challenged mice receiving vehicle control compared to those receiving exogenous rhADAMTS-13 (Figure 4C).

### 2.5. Infusion of rhADAMTS-13 Reduced Thrombosis

**Figure 5:**
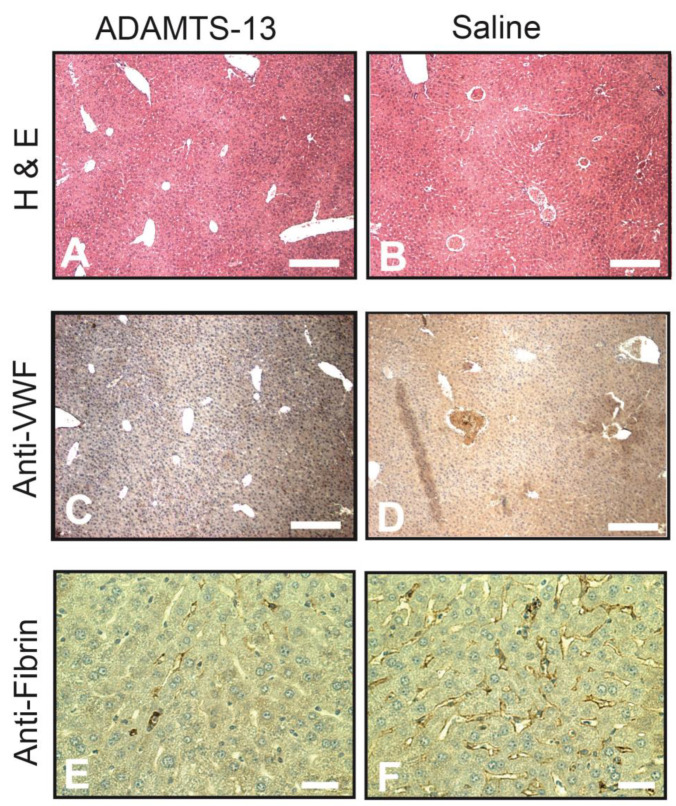
Immunohistochemistry: the liver was collected from mice sacrificed 6 h after LPS injection, sectioned, and stained for H&E ((**A**,**B**), bar = 100 µm), VWF ((**C**,**D**), bar = 100 µm), and fibrin ((**E**,**F**), bar = 20 µm) on an automatic histochemistry-staining platform. Images are representative of sections from 25 mice in each experimental group.

Mice were extensively perfused with warm PBS through cardiac puncture immediately after euthanasia to remove blood from the vasculature. Immunohistochemistry was performed on the tissue collected from mice 6 h after LPS injection to avoid bias against death, which occurred 12 h or later following LPS injection. Paraffin sections were stained for standard H&E, VWF, and fibrin. We found widespread vessel occlusion in mice receiving vehicle control after LPS injection, whereas it was reduced or undetectable in rhADAMTS-13-treated mice (Figure 5A,B). The thrombi found in these sections were stained extensively for VWF (Figure 5C,D). The liver tissue from mice receiving vehicle controls was also extensively stained for fibrin in vessels with or without thrombosis compared to those receiving rhADAMTS-13 (Figure 5E,F).

## 3. Discussion

We showed that rhADAMTS-13 significantly reduced the mortality of mice with LPS-induced endotoxemia (Figure 3). The survival improvement was more significant in female mice, but the mechanism for this gender difference remains to be investigated. There are several lines of evidence to suggest that this survival improvement is at least partially attributed to the improvement in ULVWF cleavage. First, the infusion of rhADAMTS-13 reduced thrombocytopenia. Platelet counts significantly decreased upon LPS injection and began to recover 48 h later. The increase in platelet counts was significantly greater in LPS mice receiving rhADAMTS-13 (Figure 4A). Second, ADAMTS-13 activity was deficient in mice receiving LPS but was significantly increased in those infused with rhADAMTS-13 (Figure 4C). Third, rhADAMTS-13-treated mice had fewer VWF-rich and fibrin-rich thrombi (Figure 5). Although used at a low dose of 6 mg/kg, LPS injection resulted in more than 50% death during a 7-day period after LPS injection. This mortality rate was higher than previously reported [24,25]. The exact reason for this high mortality in our model is not known but could be attributed to two factors. First, the mice used in the study were older (an average age of 10.5 weeks) than those used in other studies (8 weeks). Second, experimental results were obtained from three different batches of LPS, which induced significantly different mortality rates, ranging from 21–89%. This variation in LPS potency was overcome by using a large number of mice for the survival study. Nevertheless, these results demonstrate that the proteolysis of ULVWF multimers, which is released in abundance during systemic inflammation, is deficient or insufficient in mice with LPS-induced endotoxemia. Yet this level of proteolysis was not as severe as that of ADAMTS-13 null mice on a high VWF background [26]. These results support a previous study showing that ADAMTS-13 inhibits thrombus growth at sites of vessel injury in a mouse model [23]. They are also consistent with previously demonstrated ADAMTS-13 deficiencies in pediatric [13,18] and adult [16] patients with sepsis, a severe form of systemic inflammation.

The moderate deficiency of ULVWF proteolysis found in endotoxemic mice is likely caused by multiple factors. First, ADAMTS-13 could be consumptively deficient, as supported by the finding that ADAMTS-13 activity becomes transiently deficient in healthy subjects infused with either LPS or Desmopressin, which induces the release of ULVWF from endothelial cells [14,19,20,27]. Second, ADAMTS-13 could be proteolytically inactivated by thrombin and plasmin [22], which are produced because of an LPS-induced hypercoauglable state. Proteolytic fragments of the metalloprotease were detected in plasma samples of patients with sepsis associated DIC [16]. The excessive fibrin deposition to the vasculature further indicates that there is thrombin generation in LPS-treated mice. Finally, ADAMTS-13 activity may be directly inhibited by factors associated with systemic inflammation, such as IL-6 [28], thrombospondin [29], soluble P-selectin [30], VWF [21], and methionine oxidation [31]. Moderate ADAMTS-13 deficiency is overwhelmed by an acute surge of ULVWF secreted into circulation. Human plasma contains about 8–10 µg/mL of VWF at a resting state [32], but this VWF level rapidly increases 2–3 fold during acute systemic inflammation [33], adding 10–20 µg/mL of newly released VWF that is highly enriched in the hyperactive ULVWF to the circulation. Facing this amount of active (UL)VWF is approximately 1 µg/mL of circulating ADAMTS-13 [34], a molar ratio of more than 10 to 1. This mismatch may not be critical when proteolysis is induced after a long incubation in a static system but could potentially affect the kinetics of ULVWF cleavage in constant and rapid flowing blood. Consistent with this possibility, the cleavage of ULVWF strings under flow conditions requires higher ADAMTS-13 activity compared to that under static conditions [35]. More importantly, unlike VWF, the rate of ADAMTS-13 synthesis in the liver does not appear to be upregulated by proinflammatory cytokines [36]. Providing exogenous ADAMTS-13 could, therefore, be beneficial in correcting this VWF and ADAMTS-13 imbalance and, thus, reducing the risk of thrombosis.

In summary, we showed that rhADAMTS-13 significantly improves the survival of endotoxemic mice, primarily by improving ULVWF proteolysis and reducing thrombotic complications. These data support an early study suggesting that rhADAMTS-13 functions as an anti-thrombotic agent, potentially for complications associated with systemic inflammation.

### Study Limitations

We showed that the proteolytic cleavage of ULVWF was reduced in mice with LPS-induced endotoxemia, but the underlying mechanism responsible for this reduction remains to be investigated. For example, it is unclear, particularly in vivo, whether LPS-induced endotoxemia specifically reduced ADAMTS13 synthesis or inhibited VWF proteolysis by the upregulation of other inhibitory factors present during systemic inflammation (e.g., IL-6, thrombin, and oxidative stress). Such conclusions could not be drawn using a flow-based assay so warrant further study. In addition, the mice used in the study were not of advanced age, where thrombosis, thrombocytopenia, and mortality may be more exaggerated in response to endotoxins.

## 4. Materials and Methods

### 4.1. Expression and Purification of Recombinant ADAMTS-13

A full-length cDNA of human ADAMTS-13 was cloned into the mammalian expression vector pSectag2 (Invitrogen, Carlsbad, CA) and transfected into Hela cells (ATCC) from human cervical cancer using lipids as a carrier, as we previously described [37]. The transfected cells were grown in DMEM/F-12 medium with 10% fetal bovine serum (Invitrogen, Grand Island, NY, USA) and hygromycin (400 µg/mL, final concentration) and were sorted for high expression by flow cytometry single-cell sorting. For production, cells were grown in T-250 flasks until 90–95% confluence and then incubated with serum-free CD CHO-A medium (Invitrogen) containing 1% glutamine for 24–72 h. The conditioned medium was collected and centrifuged at 1500× *g* to remove cell debris. The rhADAMTS-13 was purified using a Y-per^TM^ 6xHis fusion protein purification kit (Pierce Biotechnology, Rockford, IL, USA) and concentrated by a Millipore spin column (Amicon Ultra-15, Billerica, MA, USA). The obtained rhADAMTS-13 had a purity of ~86%. The culture medium from sham transfected cells was similarly processed and used as vehicle control.

### 4.2. Mouse Model and Treatment Schedule

This animal study was approved by the Institutional Review Board of Baylor College of Medicine (111-02, 3 July 2016). The experiments were conducted using wild-type C57BL/6J mice (Jackson Laboratory, Bar Harbor, ME). Mice received intraperitoneal injection of polysaccharides from *Escherichia coli* 0111:B4 (LPS, Sigma Chemical, St. Louis, MO, USA) at 6 mg/kg and were infused with either 5 µg per mouse of rhADAMTS-13 (200–250 µg/kg in 100 µL volume) or an equal volume of vehicle through the tail veins 30 min after LPS injection. To determine the number of mice needed for the study, we first performed a pilot study to measure the 48 h survival rate of LPS-treated mice without treatment. We then used the survival data to calculate the number of mice needed for the study to ensure sufficient samples for blood tests during the first 72 h.

Citrated blood was collected through orbital puncture (100 µL in each draw) 24 h, 72 h, and 7 days after LPS injection for measuring platelet counts on an ABX Micros 60 blood analyzer (Horiba ABX Diagnostic, Irvine, CA, USA) and plasma VWF antigen by ELISA. The treated mice were maintained at room temperature on a regular chow diet for one week to monitor survival. Mice in a subgroup were sacrificed 6 h after LPS injection for immunohistochemical examination of the liver. We selected this time point because 100% of mice survived 6 h or longer after LPS injection, so the selection would not be biased against death and avoided detecting pseudothrombosis associated with the cessation of blood circulation upon death. Before organ collection, blood was removed from the vasculature by infusing 50 mL of warm PBS through the left atrium until outflow from the right ventricle was clear.

### 4.3. Clearance of Infused rhADAMTS-13

An ELISA-based assay and immunoblotting were utilized to determine the clearance rate of rhADAMTS-13, which has a 6 x His-tag, from the circulation. For the former, 20 µL of mouse plasma were diluted with phosphate-buffered saline (PBS) to 200 µL and incubated in Ni^2+^-coated microtiter plate (Ni-NTA HisSorb Strips, Qiagen, Valencia, CA, USA) overnight at 4 °C. After extensive washing with PBS, the captured metalloprotease was detected by first incubating plates with a goat ADAMTS-13 antibody (#156, Bethyl Laboratories, Montgomery, TX, USA) for 60 min and then with rabbit peroxidase-conjugated anti-goat IgG (Pierce Biotechnology) for 45 min at 37 °C. The bound antibody was detected by the HRP substrate TMB (Sigma, St. Louis, MO, USA) at OD 450 nm. The standard curve was generated using 0 to 240 ng of purified rhADAMTS-13. This Ni-capturing step was necessary to specifically detect the recombinant human ADAMTS-13 given to LPS-stimulated mice and to avoid cross-species reaction of the antibody used for the experiments.

To verify results from the ELISA, the ADAMTS-13 clearance rate was also determined by immunoblots. Briefly, 100 µL of citrated blood was incubated with Ni^2+^-coated sepharose beads (GE Healthcare Life Science, Piscataway, NJ) overnight at 4 °C to capture rhADAMTS-13 through its C-terminal His-tag. After extensive washes with PBS, captured ADAMTS-13 was resolved on 10% SDS-PAGE and recognized by a goat ADAMTS-13 antibody (#156, Bethyl Laboratories).

### 4.4. ADAMTS-13 Activity under Flow Conditions

Since the assay requires approximately 1 mL of blood, it was performed on a subgroup of 28 mice (14 from each group). For this, mice were sacrificed 6 h after LPS injection, and citrated blood was collected through heart puncture. Platelet-rich plasma (PRP) was obtained by centrifuging blood at 2000× *g* for 15 min at 25 °C and mixed with washed human platelets suspended in complete Tyrode’s buffer at a 1:1 ration (platelets served as a marker for ULVWF strings). The reconstituted PRP was then perfused over a monolayer of histamine-stimulated human umbilical vein endothelial cells (HUVECs) under a shear stress of 2.5 dynes/cm^2^ for 2 min in a parallel-plate flow chamber system [6,28]. Platelet-adherent ULVWF strings were counted in 20 continuous review fields (200× magnifications) after 2 min perfusion. ADAMTS-13 activity was defined as a percentage reduction in the number of ULVWF strings by perfusing treated plasma compared to untreated plasma [6].

### 4.5. Plasma VWF Antigen, Platelet Counts, and D-Dimer

Plasma VWF antigen was measured using a commercial ELISA kit (Ramco Laboratories, Houston, TX, USA) for human VWF and then validated using a commercial ELISA kit for mouse VWF (Abcam, #ab208980, Cambridge, MA, USA), all in accordance with the instructions of the manufacturer. The reason for this apparent redundancy is to ensure proper detection of ADAMTS-13-cleaved VWF because the human kit uses a polyclonal antibody, which binds VWF independently of cleavage. Platelet counts were quantified using ABX Micros 60 blood analyzer (Horiba Medical, Montpellier, France). Plasma levels of D-dimer were used as a marker for fibrinolysis and were measured using a commercial ELISA kit, in accordance with the instructions of the manufacturer (Abbexa, Cambridge, UK).

### 4.6. Immunohistochemistry

Liver sections were first deparaffinized and hydrated through a series of washing steps from alcohol to distilled water. They were sequentially stained first with Gill II hematoxylin for 3 min and then Eosin Y for 45 s. The stained slides were washed in 95% alcohol, dehydrated, cleared in xylene, and mounted using Cytoseal.

Tissue sections from the liver were also immunostained for VWF using an automated staining system (Dako Cytomation, Carpinteria, CA, USA). Briefly, antigen was first retrieved by incubating tissue sections in a sodium citrate buffer (10 mM, pH 6.0) containing 0.05% Tween-20 for 20 min. The sections were quenched of endogenous peroxidase activity by 0.3% hydrogen peroxide (15 min) and blocked with a serum-free protein blocking solution for 5 min. They were then incubated with either a rabbit anti-VWF (Agilent Dako, Santa Clara, CA USA), mouse anti-fibrin (Agilent Dako), or control IgG for 60 min at room temperature, followed by extensive washing with Tris buffer saline (pH 7.6). The sections were incubated with Envision anti-rabbit or anti-mouse HRP polymers (Agilent Dako) for 30 min, followed by 8 min incubation with DAB substrate (Agilant Dako). After being counterstained in Hematoxylin, the sections were mounted with Cytoseal.

### 4.7. Data Analysis

Quantitative data were presented as mean ± SEM and analyzed using Signa plot V12. Multiple group comparisons were made, using one-way analysis of variance (ANOVA) if the data passed the Shapiro–Wilk normality test and Kruskal–Wallis one-way ANOVA on ranks if they failed the normality test. To compare data between two groups, Student’s t-test or the Mann–Whitney rank-sum test was used depending on the results of normality tests. The survival data were evaluated by Kaplan–Meier analysis. A *p*-value of less than 0.05 was considered statistically significant.

## Figures and Tables

**Table 1:** Age and body weight of tested mice.

	Vehicle Control	ADAMTS-13
Age (weeks)		
Male	10.6 ± 0.2	10.7 ± 0.3
Female	10.0 ± 0.3	10.8 ± 0.4
Body weight (g)		
Male	25.6 ± 0.3	23.6 ± 0.7
Female	19.9 ± 0.3	20.1 ± 0.3

## Data Availability

All data included in this manuscript will be available upon publication.
